# Application of Advanced Nanomaterials for Kidney Failure Treatment and Regeneration

**DOI:** 10.3390/ma14112939

**Published:** 2021-05-29

**Authors:** Aziz Eftekhari, Solmaz Maleki Dizaj, Elham Ahmadian, Agata Przekora, Seyed Mahdi Hosseiniyan Khatibi, Mohammadreza Ardalan, Sepideh Zununi Vahed, Mahbuba Valiyeva, Sevil Mehraliyeva, Rovshan Khalilov, Mohammad Hasanzadeh

**Affiliations:** 1 Pharmacology and Toxicology Department, Maragheh University of Medical Sciences, Maragheh 7815155158, Iran; ftekhari@ymail.com; 2 Russian Institute for Advanced Study, Moscow State Pedagogical University, 1/1, Malaya Pirogovskaya St., 119991 Moscow, Russia; hrovshan@hotmail.com; 3 Dental and Periodontal Research Center, Tabriz University of Medical Sciences, Tabriz 5166614756, Iran; maleki.s.89@gmail.com; 4 Kidney Research Center, Tabriz University of Medical Sciences, Tabriz 5166614756, Iran; s.m.khatibi@irri.org (S.M.H.K.); sepide.zununi@gmail.com (S.Z.V.); 5 Chair and Department of Biochemistry and Biotechnology, Medical University of Lublin, Chodzki 1 Street, 20-093 Lublin, Poland; 6 Department of Pharmaceutical Technology and Management, Azerbaijan Medical University, AZ 1022 Baku, Azerbaijan; mahbubav_amu@rambler.ru (M.V.); sevil66@mail.ru (S.M.); 7 Department of Biophysics and Biochemistry, Baku State University, AZ 1148 Baku, Azerbaijan; 8 Institute of Radiation Problems, Azerbaijan National Academy of Sciences, AZ 1001 Baku, Azerbaijan; 9 Pharmaceutical Analysis Research Center, Tabriz University of Medical Sciences, Tabriz 5166614756, Iran

**Keywords:** kidney regeneration, therapeutic nanomedicine, exosomes, nanovesicles, carbon nanotubes, nanofibers, electrospinning, synthetic kidney

## Abstract

The implementation of nanomedicine not only provides enhanced drug solubility and reduced off-target adverse effects, but also offers novel theranostic approaches in clinical practice. The increasing number of studies on the application of nanomaterials in kidney therapies has provided hope in a more efficient strategy for the treatment of renal diseases. The combination of biotechnology, material science and nanotechnology has rapidly gained momentum in the realm of therapeutic medicine. The establishment of the bedrock of this emerging field has been initiated and an exponential progress is observed which might significantly improve the quality of human life. In this context, several approaches based on nanomaterials have been applied in the treatment and regeneration of renal tissue. The presented review article in detail describes novel strategies for renal failure treatment with the use of various nanomaterials (including carbon nanotubes, nanofibrous membranes), mesenchymal stem cells-derived nanovesicles, and nanomaterial-based adsorbents and membranes that are used in wearable blood purification systems and synthetic kidneys.

## 1. Introduction

Organ loss and/or damages induced by diseases, traumas and medical manipulation are among the frequent and disturbing health problems. Organ transplantation is the ideal option in order to replace or restore the functionality of injured tissues [1]. The annual cost of surgical producers for organ transplantations is estimated to be around 400$ billion, which encompass more than 8 million surgeries. The conventional implants applied in tissue transplantations contain autografts, allografts and xenografts [2]. The restriction in donor sites are the drawbacks in autograft procedures, but still are the most frequent types of transplantation. On the other hand, allografts and xenografts can be applied extensively, but their use is associated with elevated risk of immune rejection and disease transmission [3]. Therefore, an urgent solution is required in order to overcome these limitations. The implementation of biology and physiology in clinical practice is considered as the realm of biomedicine. The current biomedicine benefits from the progresses in tissue engineered products as promising substitutes of injured tissues/organs [1,2,3,4,5,6].

Tissue engineering, first described in 1988, is an interdisciplinary field which uses the principles of both medical sciences and engineering of biomaterials in developing biological alternatives that may replace, restore, and preserve the organ function [7,8]. It is generally based on three important constituents: cells, scaffolds and signaling molecules that enhance cell growth [9]. The suitable environmental conditions facilitate the interaction of these components in regeneration of a new tissue/organ. Scaffolds provide the favorable environment for cells, enhancing their proliferation and specified differentiation through delivering nutrients and growth factors [10]. High biocompatibility, biodegradability, mechanical stability and the ability to simulate the chemical composition and morphological structure of the extracellular matrix (ECM) are the properties of an optimal scaffold which then enhances the cellular attachment, proliferation and differentiation to a particular new tissue [4,7].

The type of biomaterials in the construction of scaffolds plays a key role in tissue engineering. Several materials, including natural and synthetic polymers, ceramics and composites, have been applied in the fabrication of scaffolds. Meanwhile, the distinct specifications of natural polymers, such as enzyme-regulated degradability, suitable biological performance and inherent cellular interactions, which all resemble the ECM have turned them into popular scaffolds used in bioengineering.

Collagen as a natural polymer is the main constituent of ECM found in bone, skin, cartilage and tendon tissues [11], and has been extensively utilized in tissue engineering. For instance, Apligraf^®^ is the artificial skin applied as a dermo-epidermal graft consisting of bilayered collagen gels which are seeded with human keratinocytes and fibroblasts [12]. Different types of collagen scaffolds such as sponge, gels and nanofibers have been used in tissue engineering of bone, cartilage and skin [13,14]. To recover the renal function after ischemia/reperfusion injury, injectable collagen hydrogel has been applied, promoting migration and recruitment of host renal stem cells and inducing in situ regeneration of renal glomerular and tubular structures [15]. Moreover, other natural compounds such as gelatin [16], silk [16], chitosan [17], alginate [18] and chondroitin sulphate [19] have been implemented in the production of scaffolds to reconstruct different tissues/organs. However, low mechanical strength, immune rejection, pathogen transmission and bench to bench variations are also considered as drawbacks of natural polymers [20].

The ultimate goal of tissue engineering is enhancing the life expectancy of patients through providing a substitute with architected organ, including the kidneys. The kidney as a vital body organ which purifies the blood and thus any disturbances in its function is attributed to potential adverse outcomes. The prevalence of chronic kidney disease (CKD) is increasing at a detrimental rate, and in spite of traditional therapies in the context of pharmacological intervention that control the risk factors, novel emergent options are being implicated as new avenues in the regeneration of diseased kidneys [21].

Renal transplantation has been considered as the most superior approach in renal failure compared to other techniques, including dialysis. However, a long time period before a suitable kidney donor is found and also fewer kidney numbers than the patients needing replacement therapy are the main obstacles. Accordingly, renal regeneration has been considered as the major problem in a healthcare system, grabbing great attention [22]. Nanotechnology, as a unique and rapidly growing field of science, has offered groundbreaking benefits in several areas, including medicine and biology. Therefore, nanoengineered biomaterials can play a profound role in renal failure treatment and kidney regeneration [21,22].

In general, nanomaterials are described as materials with any external or internal structures within the range of 1–1000 nm. Nevertheless, in nanotechnology field, nanomaterials are commonly defined to have diameter within 1 to 100 nm [23]. Nanomaterials as engineered molecules have offered an encouraging outlook with diverse superiorities [24,25,26,27]. Broad types of materials such as polymers, metals, lipid, modified macromolecules and semiconductors have been implemented in nanotechnology. Exceptional physicochemical properties of nanomaterials in relation to their size have enabled them to be used in the diagnosis, treatment and investigation of various diseases; however, a few of them particularly addresses renal pathologies. Targeted delivery of drugs to the specific cells/tissues might be the most eminent feature of nanotechnology in biomedicine. Additionally, it has enhanced drug solubility, diminished off-target unwanted effects, and has provided new diagnostic options in clinical practice. Plausible kidney-related nanomaterials are the target of increasing cohort studies in this field [21,22].

The controlled specifications of synthetic polymer nanomaterials have enabled them to be used in specific conditions [8,28]. Moreover, they possess a broad range of physicochemical and mechanical properties in the context of degradation rate, elastic modulus and tensile strength [29]. Different synthetic polymers, such as polyglycolide or poly (glycolic acid) (PGA), polylactic acid (PLA), poly lactic-co-glycolic acid (PLGA), poly (vinyl alcohol) (PVA) and polycaprolactone (PCL), have been used in tissue engineering applications to produce nanostructured biomaterials (e.g., nanosheets, nanofibers, nanocoatings) [30,31,32]. Although they exhibit several advantages, synthetic polymers yield acidic products upon degradation which might induce the inflammatory response in neighboring tissues. Moreover, the lack of recognition site in these polymers, which is crucial for cell attachment and growth, is another disadvantage. The use of natural and synthetic polymers combined has brought a potential solution for these problems by providing higher mechanical strength, cell penetration capability and a tunable degradation process [33]. Polymeric matrix of the biomaterial is also often reinforced with nanoparticles to synthesize nanocomposite material with improved biological and mechanical properties [34]. Several nanocomposite biomaterials have been produced as potential scaffolds for regenerative medicine applications. For instance, Wu et al. produced an electrospun nanofiber made of polyvinyl alcohol (PVA) and Tobacco Mosaic Virus (TMV) nanoparticles that had the ability to provide higher cell density of baby hamster kidney cells [35]. Whereas osteochondral defects have been successfully treated via hydroxyapatite/chitosan [36] and PLGA/collagen [37] nanocomposite scaffolds. Furthermore, the application of decellularized matrix has been a breakthrough innovation in simulation of ECM and contains several types of biopolymers needed in regenerative medicine [38,39] of different tissues, such as small intestine [40], heart valves [41] and urinary bladder [42].

Different nanosized therapeutic agents are now being evaluated in clinical trials, and many of them have been approved in cancer theranostics. For instance, 99mTc-labeled sulfur colloids and superparamagnetic iron oxide nanoparticles have received approval for liver metastasis and lymph node imaging. Optical imaging should be possible as a result of ongoing trials using Cornell dots (Cdot) with effectual renal clearance [43]. Thus, increasing translational experiences generate a potential platform for sequential progress of new nanomedicines in the diagnosis and treatment of renal diseases.

The balance between renal and hepatobiliary excretion of nanoparticles remains to be understood comprehensively [44]. Moreover, nanomaterials with different sizes have different segment target of the renal system [44]. Bulk production of nanomedicines is also required in order to simplify the clinical management of kidney-related diseases which in turn not only need high standard synthesis methods but also demands safe assessments in human [45].

Electrospinning is a versatile, robust and cost-effective method for fabrication of nanofibers. Ebara et al. used this method for the production of nanofiber mesh with incorporated silicon and aluminum. The silicon/aluminum ratio determined the creatinine adsorption level [46]. Although their product is still a prototype, it might appear to be a suitable alternative of dialysis. Similarly, researchers have developed a novel nanofiber mesh to remove toxins from the circulation [47]. Since the kidneys have a natural ability to clear particles (<12 nm), they are considered as outstanding targets of nanoparticles (NPs). The function of kidney results in the excretion of particles below 2 nm, decreased clearance at 6 nm and no renal excretion of particles larger than 11 nm [44,46].

The combination of nanotechnology, tissue engineering and material sciences has promising applications in the biomedicine. The exponential progress in these emerging fields has vividly improved the quality of human life to a great extent. The bedrock for the synthesis of novel substrate for the regeneration of the kidney is a deep understanding of the features of different biomaterials. Consequently, presented review focuses on the implementation of advanced nanomaterials (carbon nanotubes, nanofibers, cells-derived nanovesicles) for kidney failure treatment and regeneration, including nanomaterial-based adsorbents and membranes that are used in wearable blood purification systems and synthetic kidneys.

## 2. Kidney-Targeted Delivery Systems

Targeted therapy in renal system not only can enhance the efficacy of drugs but also diminishes toxicities. The functional unit of kidney, known as nephron, consists of the glomerulus and tubules. The glomerulus which is composed of blood capillary tufts and the mesangium contains the compartments of glomerular filtration barrier (glomerular endothelial cells (GECs), glomerular basement membrane (GBM), and podocytes) [43]. The filamentous structure of GECs prohibits the entrance of plasma constituents into the membranes of endothelial cells. In the next layer, components such as collagen IV, nidogen, laminin and proteoglycan are found and form a tick connective tissue membrane [48,49]. Podocytes produce filtration slits via their interdigitating processes [50].

Prodrugs, macromolecular carriers and nanoparticles have been the most prevalent approaches in kidney-targeted drug delivery systems [51,52,53,54]. The function of renal enzymes results in the release of the active form of prodrugs as a selective renal targeting system [53]. Different peptides, proteins, viruses and antibodies have been applied as macromolecular carriers of kidney-targeted therapy [52]. Additionally, nanomaterials have exhibited excellent potential in this context [54].

### Kidney-Targeted Drug Delivery Systems Based on Nanoparticles

Nanoparticles have been applied as novel means of renal disease theranostics. Targeting kidney via nanoparticles is due to their unique properties via tailoring size, shape, charge and ligands. Moreover, nanoparticles are promising tools of fabricating implantable artificial kidneys [55]. Exposure to different classes of nanoparticles may have undesirable effects on cells and organs. A size-dependent solute rejection is observed in nanoporous membranes due to their filtration features. These groundbreaking progresses in membrane technology provides possibility to develop potential implantable renal replacement options [56].

Nanoparticles can cross biological barriers and reach their target cell because of their physiochemical properties. Nanoparticles with diameter of 75 ± 25 nm can be designated to renal mesangium, showing therapeutic effects [57]. Actinomycin D (AD)-loaded isobutyl acrylate nanoparticles (ADNP) have been successfully concentrated in mesangial cells and exhibited beneficial effects both in vitro and in vivo in an empirical glomerulonephritis model [54].

Liposomes are considered as well-known drug delivery systems. Small unilamellar vesicles (SUVs)-loaded methotrexate [(MTX)SUVs) connected with monoclonal antibody has been used in treating human renal cancer via targeting cell proliferation [58]. A liposome-based system containing Fab fragments of OX7 mAb has been constructed to target Thy1.1 antigen in mesangial cells [59].

Nanoparticles not only act as drug carriers but also show promising effects as effective drug candidates. However, it should be considered that drug-carrying nanoparticles could attach directly to the lipid membrane due to premature release of encapsulated drugs, causing toxicity to cells.

High thermal and biological stability, simple preparation, versatility are advantages of inorganic nanoparticles, which have turned them into excellent compounds in biological, chemical and drug industry systems [60]. Recent studies have shown the positive effects of inorganic nanoparticles in oxidative-stress-liked diseases including acute kidney injury AKI [61,62]. Molybdenum-based polyoxometalate (POM) nanoclusters have shown superior renal uptake and subsequently antioxidant effects in kidney during AKI [63]. Similarly, selenium nanoparticles have improved AKI via antioxidant, anti-inflammatory and anti-apoptotic effects [64]. Porous Se@SiO_2_ nanospheres have also inhibited oxidative damage through direct and indirect pathways [65]. The delivery of siRNA as well as multimodal imaging has been developed using superparamagnetic iron oxides and indocyanine green in a PLGA matrix and the surface was coated with polyethyleneimine [66].

**Figure 1:**
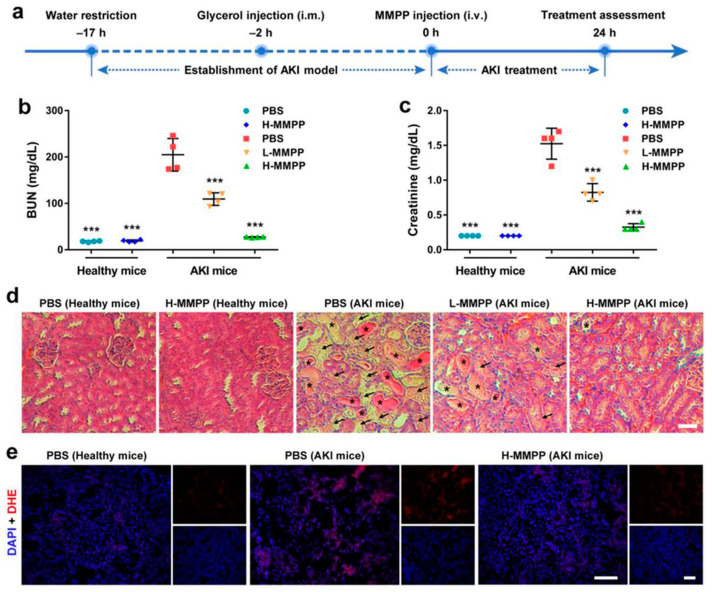
Induction of acute kidney injury (AKI) in animal models and the role of polyethylene glycol-incorporated (PEGylated) Mn^2+^-chelated melanin nanoparticles (MMPP NPs): (**a**) Schematic diagram of the formation of an animal AKI model and their treatment with MMPP nanoparticles; (**b**,**c**) kidney function in normal and AKI mice treated with phosphate-buffered saline (PBS) or MMPP nanoparticles; (**d**) H&E-staining of renal tissues from healthy and AKI mice treated with PBS or MMPP nanoparticles; (**e**) confocal images of dihydroethidium (DHE) and DAPI-staining of renal tissues from normal and AKI mice treated with PBS or MMPP nanoparticles. *** *p* < 0.001 in compared with PBS-treated AKI controls. Reproduced with permission [67]. 2019 WILEY-VCH Verlag GmbH and Co.

Figure 1 illustrates the protective role of ultrasmall Mn^2+^-chelated melanin nanoparticles incorporated with polyethylene glycol nanoparticles (MMPP NPs) in the mitigation of renal ROS formation during AKI in vivo. The results demonstrate the possible antioxidant effects of these NPs [67].

## 3. Nanoparticles and Nanomaterials for Kidney Regeneration

Different nanoparticles and nanomaterials have been implemented in the regeneration of renal tissue via the aid of novel technologies which will be discussed in the following sections.

### 3.1. Carbon-Based Nanomaterials

Carbon nanotubes (CNTs) as tube-shaped nanomaterials are made from graphite sheets which determine their metallic or semi-conductive nature [68]. CNTs are fabricated with three major techniques: (1) Laser ablation, (2) chemical vapor deposition and (3) discharge and are classified into two main groups: single-wall carbon nanotubes (SWCNTs) and multiwall carbon nanotubes (MWCNTs) [69,70,71,72,73,74]. CNTs have been applied to overcome limitations of some scaffolds. For instance, reinforcement of a collagen scaffold with CNTs having the favorable specifications has enhanced its mechanical properties [75,76]. Moreover, the combination of CNTs with synthetic biocompatible polymers has been commonly used in tissue regeneration. A composite of MWCNT and poly (L-lactide) has increased the crystallization, palletization and conductivity of the polymer as well as decreased its growth inhibitory effects on fibroblasts [77]. Encapsulation of MWCNTs in the PLA nanofibers has resulted in a novel electrospun nanocomposite with increased conductivity and plummeted fiber diameter in comparison with pure PLA. Additionally, the growth of adipose-derived human mesenchymal stem cells (hADSCs) has shown an upward trend on this composite [78].

**Figure 2:**
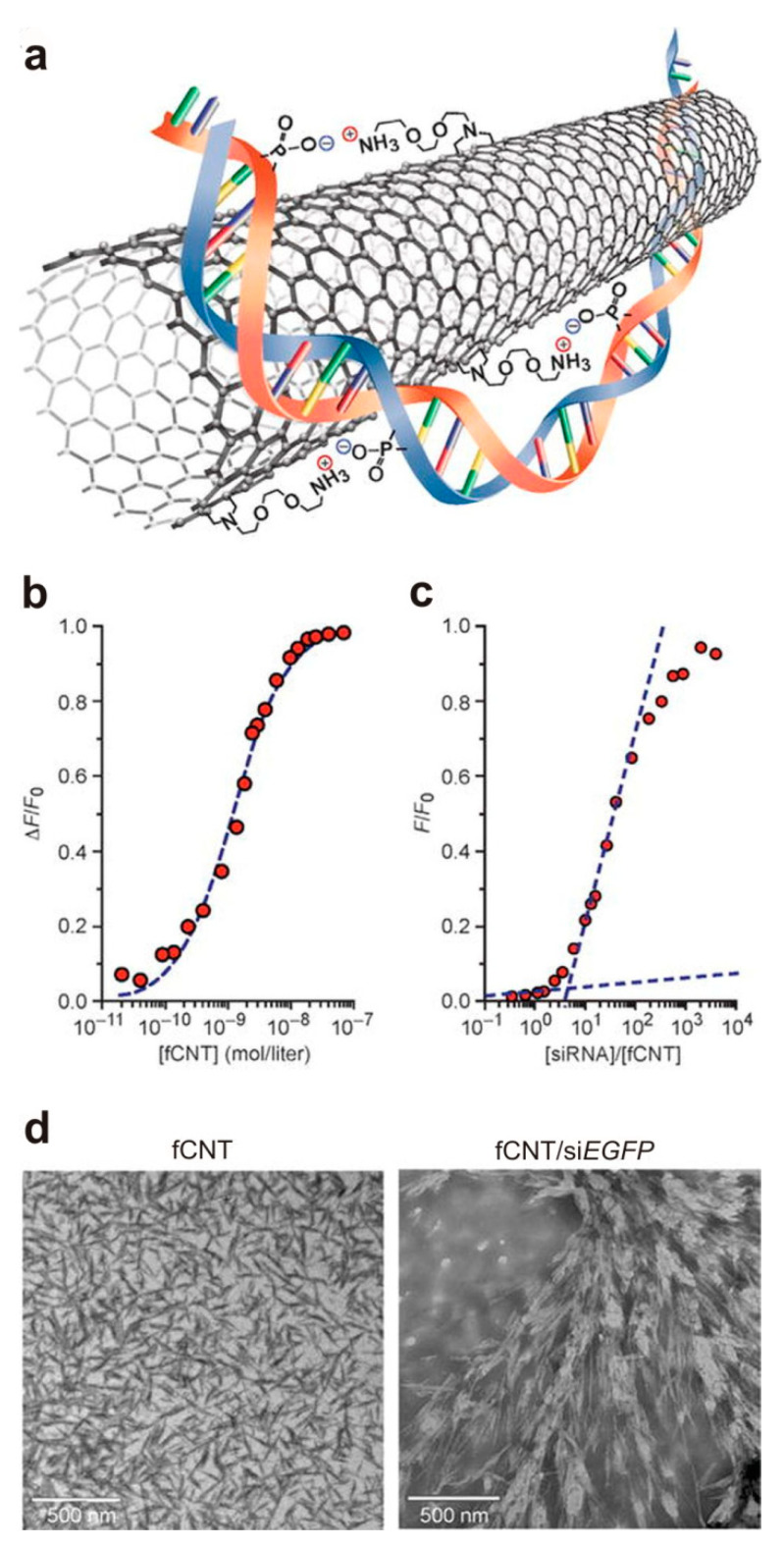
Assembly of the CNT siRNA structure: (**a**) A representation of the noncovalent bonding interactions between siRNA and fCNT; (**b**) The fluorescence quenching titration of siEGFP-Cy3 with fCNT and fitted binding isotherm (dashed line); (**c**) Relative fluorescence intensity as a function of siEGFP-Cy3/fCNT molar ratio and graphical interpolation of the curve; (**d**) TEM images of solid-state fCNT and fCNT/siEGFP (1:1 complex). Reproduced with permission [87]. Copyright 2016, American Association for the Advancement of Science.

Nanotubes have the capability to be handled using miniature needles and to transfer from cellular membrane through an automatic pathway with an unidentified mechanism. However, there are some toxicity concerns regarding some nanotubes including arc-discharge single-walled carbon nanotubes that need to be understood before their biomedical applications [79]. In general, it is suggested that functional groups and metal catalyst impurities may cause the toxicity of carbon nanotubes [80]. According to computer simulations, functionalized CNTs accumulate onto the membrane space in a parallel manner to the membrane sheet. These materials are able to enter different cell types alike human T-cells and promyelocytic leukemia cells. The mentioned functions can also be achieved in kidney cells, assisting renal tissue regeneration. Both therapeutic and diagnostic agents could be absorbed/attached on the plane of CNTs after encapsulation [81]. In this context, heparin-based CNTs have been estimated to pose great significance due to their good blood compatibility. The veneering capability of heparin or its deposition onto CNTs is the excellent properties of this anticoagulant that in this form may exhibit a similar structure to artificial kidney. Importantly, it has been shown that heparin composite membrane containing nanopores could be implemented as synthetic kidney and/or a dialysis apparatus filtering the blood and preserving its flow. Therefore, the application of a dialyzer which encompasses blood compatible CNTs might be a prominent substitute of heparin during kidney dialysis [82]. Reddy et al. examined the potential cytotoxicity and general mechanism of MWCNTs in human embryonic kidney cell line. Although the results indicated toxic effects of these nanotubes in human embryonic kidney cells, and more research is required in order to prove the acceptability of CNTs in kidney regeneration, they are currently the most used nanomaterials in this context [83]. Another important application of CNTs have been reported in the delivery of siRNAs [84]. Different drugs, radioisotopes and proteins have been delivered via ammonium-functionalized single-walled carbon nanotubes (fCNTs) [85]. The renal glomerular filtration and clearance of fCNTs were favorable in spite of large aspect ratio of these particles [86]. The proximal tubular cells partially reabsorbed filtered fCNTs which enabled them to transport non-covalently bound siRNA (Figure 2).

Therefore, in order to obtain excellent results in the application of CNTs in tissue engineering, their biodistribution and impact on other organs should be cleared out. The synthesis and development of CNTs with maximum safety on human being is deemed to be possible since their physicochemical specification contributes to their toxicity [88,89].

### 3.2. Endagenous Nanosized Structures

Exosomes as nanosized (30–100 nm) extracellular vesicles (EVs) are formed through the fusion of multivesicular bodies with the endosomal membranes and a further secretion into the extracellular space [90]. Exosomes are usually harvested from conditioned medium from primarily mesenchymal stem cell cultures. Importantly, EVs contain various biomolecules (e.g., proteins, lipids, RNA, DNA) that transfer the signals for recipient cells, inducing appropriate physiological responses [91]. EVs called cell-engineered nanovesicles may also be generated by mechanical disruption of plasma membranes [92]. A great body of evidence supports the theory that paracrine/endocrine effects of stem cells play a pivotal role in repairing the damaged tissues [93]. Several in vivo studies have confirmed that the secretomes of mesenchymal stem cells (MSCs), which encompass different growth factors, cytokines, vesicles and exosomes, are responsible for their beneficial effects [94]. The administration of conditioned medium from MSCs has increased the proliferation of renal tubular cells and decreased their apoptosis in a kidney injury induced by toxic agents [95].

Exosomes exhibit lower immunogenicity in comparison with the originated stem cells [96,97]. A great biological tolerance is also well detected in exosomes, which is an essential factor in therapeutic applications [98]. Besides, the rapid transfer of exosomes into the target cells turn them into excellent delivering components [99]. MSC-derived exosomes and nanovesicles have successfully accelerated renal tubular cell proliferation and inhibited the occurrence of apoptosis [100]. Bruno et al. for the first time has shown the protective role of MSC-exosomes in glycerol-induced acute kidney injury (AKI) [101]. The current research has investigated the mechanisms associated with these beneficial effects and has determined the role of genetic materials transferred by exosomes [102,103]. Bone marrow-derived exosomes encompass mRNAs that can induce the activation of new cell cycles in injured tissues [101]. The recovery of renal tissue has been observed after the transfer of human IGF-1 receptor mRNA in nanovesicles derived from MSCs into the tubular cells [104].

**Figure 3:**
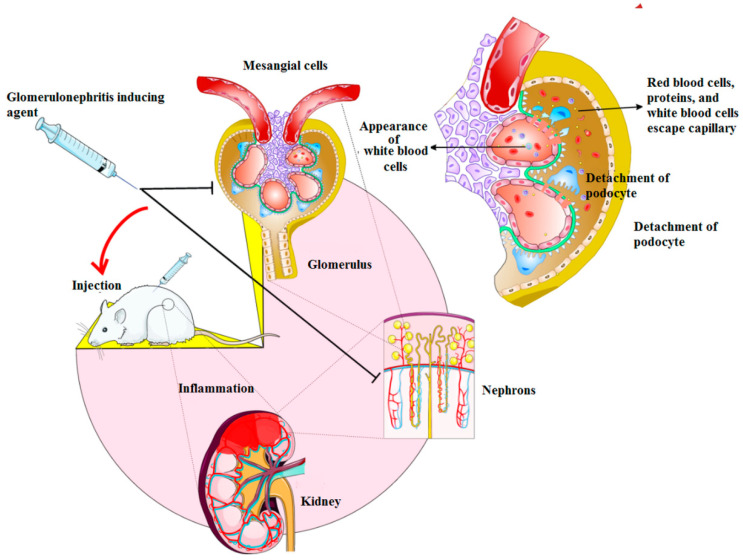
The protective role of endothelial progenitor cells (EPC)-derived nanovesicles in an experimental model of glomerulonephritis. These nanovesicles can inhibit mesangial cell proliferation, inflammatory response and proteinuria in the induced glomerulonephritis in vivo.

Recent studies have investigated more about the role of stem cells-derived exosomes [6]. The potential regeneration of kidney with vesicles isolated from human liver stem cells has been also examined. Diminished urea and creatinine level was observed three days after the induction of AKI in a murine model [105]. Similarly, in a rat model of AKI, the role of stem cells-derived vesicles was evaluated through two different tests [103]. The intravenous administration of wild-type MSCs and related vesicles has been also recently studied [106]. The improvement of AKI in the context of both structural and functional aspects was exhibited when wild-type MSCs and correlated vesicles were administrated, proposing the pivotal role of microRNA in the regeneration of injured kidney with the use of exosomes. This was in line with the results presented in the literature on the impact of extracellular vesicles in kidney renewal [103]. Moreover, Camusssi et al. showed the benefits and potential of exosomes in the treatment of damaged kidney by endothelial progenitor cells (EPCs)-derived extracellular vesicles. It can be noted that the secretion of nanovesicles is the main advantage of exosome usage [107]. It has been shown that the intravenous infusion of nanovesicles into the site of mesangiolytic glomerulonephritis in vivo results in the generation of vesicles juxta-injured areas, particularly the glomeruli. Activation of mesangial cells, cell infiltration and apoptosis machinery were inhibited in this study, which further reduced the proteinuria and elevated the hemolytic activity of serum (Figure 3). Besides, EPC-derived exosomes can protect the kidney from ischemia-reperfusion injury through tubular cells and peritubular capillaries, providing an additional way to prevent the decline in capillary density in glomerulosclerosis and tubulointerstitial fibrosis. Nevertheless, this defensive mechanism is absent subsequent to the inhibition of miR-126 and miR-296, which regulate proangiogenesis. These outcomes are valid causes for application of microRNA-based exosomes in renal therapy [108].

Accordingly, there is an increasing focus on the role of stem cells-derived exosomes in the treatment of acute and chronic renal failure as promising opportunities. A large number of empirical studies have now confirmed the pro-regenerative role of exosomes in AKI while their role in chronic kidney disease (CKD) requires further in vivo tests. Exosomes could also be applied as valuable vehicles in the delivery of nucleic acids, proteins and small drug in regenerative medicine.

### 3.3. Biomimicking Nanostructures

Biomimetics are composed of natural systems, elements and/or models in resolving human problems [109] and have opened new avenues in biological system at mainly macro and nanoscales. The concept of biomimetics was developed during the 1950s, taken from nature [110]. Although most of the applications were at the macromolecular level, a novel groundbreaking progresses in nanotechnology has shifted the researches to the nanoscale level [111]. The identification of novel techniques, such as atomic force microscopy (AFM) [112] and scanning tunneling microscopy (STM) [113], has enabled scientists to investigate the nanostructures found in the nature.

Biomimicking structures display an encouraging outbreak in tissue regeneration. Nanofibers, which are biomimicking structures having the ability to mimic extracellular matrix (ECM), can be fabricated with different techniques such as wet spinning and electrospinning. The unique structure of nanofibers (fibers with thickness of below 100 nm) has turned them into attractive tools in biomedicine [4,5,6,114,115,116].

Electrospinning is among the most convenient methodologies in yielding ECM-like structures. It has been useful in fabrication of a fibrous membrane and to evaluate its applicability in human tubular epithelial cell culturing in order to ultimately achieve a membrane to be used in bio-artificial kidney [4,5,115]. The initial study which used the fiber PCLdi(U-UPy) membrane in the regulation of a synthetic kidney was conducted by Dankers et al., who have prepared fibrous and supramolecular meshes by electrospinning method. The resultant product contained oligocaprolactone functionalized with twofold urea-UPy (U-UPy) components at both terminals of the 2 kDa oligomer. The fabricated fibers showed diameters of 0.1–1 µm and a thickness of 10–30 µm. Commercial polycarbonate (PC) membranes were then prepared with a thickness of 7–22 µm and pore dimensions of 0.4 µm. The efficiency of the supramolecular membranes was connected to the various, limited conferred features of the supramolecular polymers [117]. Importantly, formation of proximal tubular epithelial cell (PTEC) monolayers on microporous commercial PC membranes occurred with one-week delay compared to the fibrous supramolecular PCLdi (U-UPy) membranes. Moreover, gene expression assessment revealed that PTEC layers on the surface of fibrous membranes had better ability to maintain their renal epithelial phenotype. Thus, both two-dimensional and three-dimensional cultures used in the regeneration of renal tissue could be endowed from supramolecular membranes [117].

Therapeutic strategies aiming for renal replacement as well as the nephrotoxicity evaluation of kidney implementations could be used as synthetic kidney membranes, along with the use of functional and differentiated human tubular epithelial cells [118]. To preserve the cell viability, the natural niche of these cells should be replicated. Bioactive peptides and ureido-pyrimidinone (UPy)-functionalized polymers were utilized to mimic natural basement membrane through electrospinning approach. The resultant membranes exhibited a hierarchical fibrous basement membrane-like structure with self-assembled nanofibers in the electrospun microfibers. Human kidney-2 epithelial cells (HK-2) were cultured on the basement membrane simulators under usual organ conditions in a custom-built bioreactor, which enabled in situ monitoring and induction of the functionality of the culture. Microscopic investigation was performed in order to evaluate cellular viability and transmembrane leakage of fluorescently labeled inulin, determining the integrity of cell barriers. Moreover, HK-2 cells maintained a polarized cell sheet and showed a parallel gene expression profile for the proteins involved in membrane transporter system and the metabolic function of brush border enzymes, when a fresh culture medium was continuously added for three weeks in the bioreactor [118,119]. The morphological features of natural basement membrane was simulated through the hierarchical fibrous structure of the UPy-polymer-based biomaterial with micro- to nanosized designs [118]. As an important note, the formation of naturally occurring nanotopographic structures in extracellular matrices can send signals to neighboring cells, which in turn alter the cellular behavior [119,120].

To improve the functionality of the scaffolds, cumulative attention has been pointed towards in vitro and in vivo study to attain ideal scaffold design. Nanobiomimetics undoubtedly possess the most appropriate physicochemical and biological properties to be used as scaffolds in kidney regenerative medicine applications.

### 3.4. Nanofibrous Membranes for Wearable Blood Purification Systems

**Figure 4:**
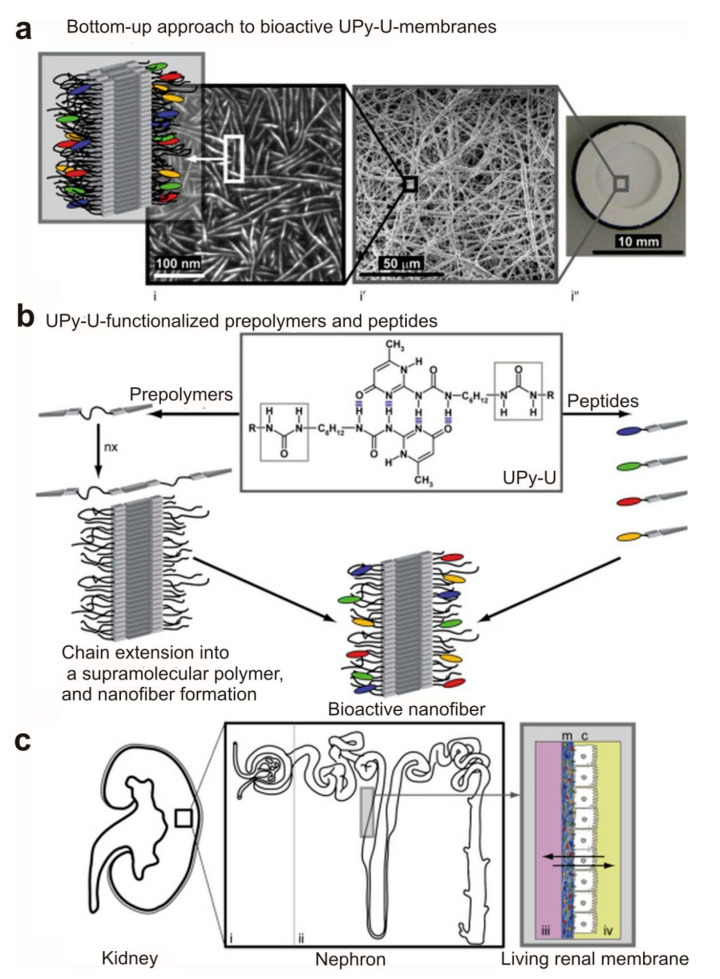
(**a**) Bottom-up method to generate bioactive UPy-U membranes; (**b**) dimers are produced via Fourfold hydrogen bonding ureido-pyrimidinone (UPy) moieties and load in lateral manner through extra hydrogen bonding in the middle of urea (U) functionalities; therefore, turning into nanofiber structures; (**c**) the representation of kidney and its different segments. Reproduced with permission [123]. Copyright 2010 Elsevier Ltd.

Microelectromechanical systems (MEMS technology) as well as the application of biocompatible polymeric nanofibers have opened new avenues in construction of a synthetic kidney. The hydraulic pressure gradients and end-to-end diffusion filtrate the metabolic toxins in artificial kidney replacement systems which utilize semipermeable membrane-based hemodialyzers [120]. Nevertheless, this system has distinct restrictions regarding the efficient filtration of by-products such as urea and creatinine. Polydimethylsiloxane (PDMS)-based artificial renal microchips which filter out the blood from poisonous materials have been synthetized using biocompatible polymeric nanofibers. Optimization of the PDMS microfluidic channel system and various packing of nanofibers membrane offered a portable and wearable artificial kidney [120]. Uremic toxins could be adsorbed using nanoporous polymers and zeolite matrix membrane combinations [121]. The applicability of polymer membranes with zeolite fillers has been proven in gas separation as well as water purification methods [122]. Electrospinning is an appropriate technique for the production of nonwoven nanofibrous membranes regarding the use of mixed zeolite powders since it produces membranes with high porosity, high surface-to-volume ratio, enhanced permeability, good size and fine interconnected pore structures [4,5]. Combinations of zeolites with electrospun polyacrylonitrile (PAN) polymeric nanofiber membranes have been used to fabricate a dialysis membrane with ideal mechanical and thermal properties, antibacterial effect, photo irradiation and improved membrane-forming specifications compared to pure porous PAN membrane. Supramolecular membranes have been utilized as 2D and 3D processes in the engineering of renal tissue. In this context, bioactive fibrous supramolecular PCLdi (U-UPy) membranes have been synthetized via the bottom-up method (Figure 4) [123].

**Figure 5:**
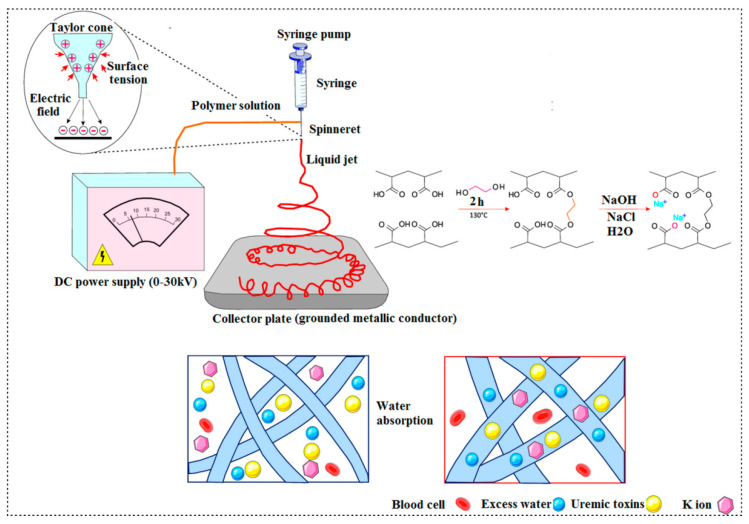
The synthesis and function of nanofiber meshes utilized in wearable blood purification systems.

The restricted available space has led researchers to fabricate micro- and nanomachining methods in artificial organ replacement therapies. Different types of artificial organs have been developed so far [124,125]. The primary model for hemoperfusion in an artificial device was introduced in 1964 [32]. The extra-corporeal removal of toxins from the human blood in which an adsorbent biomaterial (in particular activated carbon) is incorporated is the basis of hemoperfusion [33]. In spite of the applicable and valuable use of hemoperfusion in acute poisoning, its role as an artificial kidney remains challenging. There is now a rapid progress in the development of artificial kidney models [34]. There are now clinically applicable PAN membranes as dialyzer membranes, which have been made with the conventional phase inversion technique [126]. In a research conducted by Lu et al., nanofiber PAN membrane and composite PAN-zeolite membranes (composed of two zeolite types, 840-NHA and 940-HOA) were synthetized via electrospinning method using different concentrations of zeolites. Then, the team measured the capacity of both zeolite-free and zeolite-enriched membranes in the adsorption of creatinine. According to their results, a 10 wt% zeolite-enriched membrane exhibited the highest adsorption capacity [127]. It was demonstrated that the 840-NHA and 940-HOA zeolite specifications, such as the surface area and the particle size, played a key role in functional effects of the membranes in adsorbing creatinine. In another study, a wearable blood purification system without any specified apparatus was fabricated using nanofiber meshes designed with zeolite–polymer composite. Poly (ethylene-co-vinyl alcohol) (EVOH) was utilized in the structure of composite nanofibers which not only as a primary polymer matrix inhibited the release of zeolites into the bloodstream, but also as a biocompatible material enhanced the creatinine adsorption capability of the system. Composite fiber meshes could be easily obtained through the electrospinning method. Moreover, a large surface area, high porosity and the capability to be manipulated as a bulk matter due to macroscopic properties are the advantages of nanosized zeolite-polymer composite fibers. In another study, Tsuge et al. fabricated excellent water adsorbent nanofiber meshes in order to eliminate excess fluids from the circulation in chronic renal failure patients to be used in wearable blood purification system without extra apparatus. Electrospinning was used to synthetize the nanofiber meshes from poly (acrylic acid) (PAA) which was thermally crosslinked followed by neutralization of its carboxylic acid form (PAA) to sodium carboxylate form: poly(sodium acrylate) (PSA). The obtained PSA nanofiber meshes showed a great surface area and higher swelling compared to PSA film due to the presence of higher capillary forces compared to the PSA gel (Figure 5) [128]. Although composite fibers are still not fully sufficient in adsorption ability, they are proposed as potential wearable blood purification systems in distinct situations [129].

### 3.5. Nanomaterial-Based Adsorbents for Artificial Kidney

Biofabrication might be represented as the use of engineering and data sciences for automated robotic bioassembly of living 3D human tissue and organs [130]. The implantation and integration of a man-made medical devices to a human in order to replace the existing organs and maintaining their functionality is the art of artificial organ fabrication which supports the normal life of the patients as possible [124].

Novel technologies that utilize miniaturized hemodialysis models have successfully resolved some complications associated with hemodialysis such as patient movements, and problems related to its continuous, bulk and long-term application. The beneficial effects of nanoporous biological materials used in wearable artificial kidney and their essential role in adsorbing and removal of toxic urea during the dialysis process in a miniaturized system should be taken into account [35].

The closed-system, self-regenerating dialysate is one of the most important wearable artificial kidney in which the toxins must be removed from the dialysate and nitrate nanoparticles should be adsorbed with a high adsorption valence. The Guar wearable artificial kidney which uses activated carbon in its structure is now under investigation [36]. Peritoneal dialysis could also be categorized in mobile artificial kidney approaches [37] and it should be noted that peritoneal dialysis is currently among the greatest wearable artificial kidneys [38]. This system has some distinct advantages including the absence of mobility restrictions and no requirement to go to hemodialysis centers [39]. Although the implementation of nanoporous biomaterials as adsorbents is crucial in this equipment, it is still a mobile system in patients undergoing peritoneal dialysis [40]. Nanoporous (with nano-dimension porosity below 100 nm) materials as valuable adsorbents have been proven to be ideal for fabrication of artificial kidney [41,42].

Charcoal is one the first adsorbent which has been utilized in removing uremic toxins and opened the avenue for the discovery of novel adsorbents in this area [43]. Activated carbon with superiorities such as high surface area, chemically inert surface, high porosity and rapid adsorption capability has been the leading material in construction of synthetic kidney [44]. The European Uremic Toxin (EUTox) has classified the uremic toxins into three major categories: small water-soluble agents (e.g., urea, uric acid, creatinine), middle molecules (e.g., peptides, leptin), and protein-bound compounds (e.g., indoxyl, sulfate p-Cresol) [45]. The current hemodialysis systems have been classified into two main groups according to the integration of the nanoporous adsorbents. The direct contact of the adsorbent with the blood occurs in the first group which is implemented in the hemoperfusion system, while a semipermeable membrane adsorbs the uremic toxicants during dialysis through the use of nanoporous materials [46,47]. The nanoporous biomaterials integration from these two classes is made plain. Adsorbents could be represented in two groups: nanoporous adsorbents in a miniaturized hemodialysis system and nanoporous adsorbents for hemoperfusion. Each of these techniques has their own benefits and drawbacks. Nanoporous biomaterials are operated and developed in two different systems, both hemodialysis and hemoperfusion could also be used concurrently [48]. The use of activated carbon has significantly reduced the problems with renal diseases and also hemodialysis [49]. The recent research has focused on development of other nanoporous material such as zeolites to be used in artificial kidney [84]. Zeolites can adsorb low molecular weight uremic toxins with a capacity of 100 mg/kg [50]. This amount for carbon is estimated to be 150 mg/kg. Therefore, zeolite exhibits a lower and/or equal adsorption capacity to activated carbon [51].

Although efforts have been put into construction of macro and nanomachining methods, their application in macro level is still not clear because nanotechnology cannot reach the size of the body organs. However, the current approaches might provide new insights into the implementation of nanotechnology in biofabrication of human organs, including kidney.

## 4. Conclusions and Future Perspectives

The simultaneous application of regenerative medicine and nanotechnology can be an ideal approach in tissue regeneration, since the current studies show that novel emergent techniques can entirely regenerate damaged tissues. Importantly, some naturally occurring nanostructures like exosomes and cell-engineered nanovesicles have been recently proved to significantly accelerate renal regeneration. Thus, these endogenous nanostructures may be applied in regenerative medicine to enhance success rate of kidney failure treatment. Nonetheless, in spite of excellent progress that has been achieved in the realm of kidney regenerative medicine, there are still several hurdles and problems. The use of nanobiomaterials as technological drivers of innovation could pave the way in complete regeneration of kidney in the near future since the last decade witnessed evaluation of several materials and construction methods in the field of tissue regeneration, particularly the kidney. Moreover, the search for nanoengineered biomaterials which resemble the native structure of the kidney is continued to provide superior alternatives. On the other side of the coin, investigations regarding the specifications and constructions of the tissues have exhibited that the implementation of nanotechnology and/or nanobiomaterials can have a substantial effect on regenerative medicine. Accordingly, researchers propose the application of nanomaterials in renal tissue regeneration. For instance, the progress in organ-on-a-chip has been a profound development in regenerative medicine. In the recent years, the kidney-on-a-chip technology involves application of microfluidic devices mimicking the nephrons and other kidney cells and acting as an artificial substitute for the treatment of intrinsic acute kidney failure.

Nanotechnology implementation in tissue engineering processes has brought potential ideas to revolutionize the realm of regenerative medicine. However, the remaining challenge is the fabrication of suitable nanomaterials in transferring signals to the injured tissues in order to stimulate the regeneration process.

In addition, the development of a scaffold which exactly mimics the natural ECM is still a matter of question. In this context, identification of novel polymer matrices and nanomaterials with enhanced biomimicking properties is cardinal. Another concern is related to the safety of nanomaterials applied in regenerative medicine which need extensive preclinical tests. Ultimately, in order to use these nanomaterials in point-of-care settings a close cooperation between researchers and clinicians is required to determine the underlying mechanisms of interactions between the cells and applied biomaterials.

## Data Availability

Not applicable.
